# Report of a Case of Myoclonus Associated with Unilateral Epileptoid Convulsions

**Published:** 1911-11

**Authors:** James W. Putnam, Edward A. Sharp

**Affiliations:** Professor of Nervous Diseases, University of Buffalo; Consulting Neurologist, Craig Colony for Epileptics; Buffalo, N, Y.


### BUFFALO MEDICAL JOURNAL

VOL. LXVII. NOVEMBER, 1911. No. 4

### ORIGINAL COMMUNICATIONS

### Report of a Case of Myoclonus Associated with Unilateral Epileptoid Convulsions

By JAMES W. PUTNAM, M.D.
Professor of Nervous Diseases, University of Buffalo
and
EDWARD A. SHARP, M.D.
Consulting Neurologist, Craig Colony for Epileptics
Buffalo, N. Y.

THE following case of myoclonus associated with unilateral epileptoid convulsions is reported owing to the infrequency of such cases.

In 1881 Friedreich described under the title of Paramyoclonus Multiplex, a syndrome characterized by clonic spasms affecting chiefly the trunk and extremities, the spasms being short, lightning-like and involving individual muscles and not synergic muscle groups.

Later Unverricht described a form of myoclonus occurring in several members of a family and associated with epilepsy.

The first case of myoclonus associated with epilepsy reported in this country was by Clark, in 1899. The patient was at the Craig Colony, and one of the present writers had the opportunity of studying the case for several years, as well as two others of the seven cases already reported from that institution, and two additional cases which have not yet been reported.

As the bibliography has been so completely presented in the articles by Clark, Lundborg, Hunt, and others, it would be superfluous to repeat it here.

The present case was first seen by us on June 1, 1911, having been referred by the patient's physician, Dr. P. H. Whalen, of Buffalo.

The case differs from most of the recorded cases in the unilateral distribution and infrequency of the epileptoid convulsions.

H. C., male, age 22 years. Family history negative as regards nervous or mental disorders.

Patient had been nervous as a child but otherwise healthy and had devoted considerable time to athletics up to 1905, when the present trouble began.

In 1905, while wrestling, the right arm suddenly became stiff and straight and was followed by jerkings and twitchings in the right arm and leg. There was no loss of consciousness and he recovered completely in a few seconds.

The second attack occurred a few weeks later while he was in a tree picking apples. The right arm began to stiffen and shake and consciousness was lost for a few minutes. Was prevented from falling from the tree during the attack by his companion.

The third attack occurred in 1906, while he was climbing to a mountain spring. Right arm became stiff and trembling and consciousness was lost.

The fourth attack occurred in 1909 while he was home. Attack was limited to right side and there was complete loss of consciousness.

The fifth attack of the same character as the previous ones occurred a few weeks later while he was swimming in shallow water and he was rescued by his companions.

The sixth attack occurred in May, 1911, and was observed by his mother. While going down stairs the right arm began to jerk and he was able to call for help before consciousness was lost. The right arm and leg became stiff and motionless, but there was no clonic convulsion observed on the right side. The left extremities were not involved.

The seventh and last attack occurred while patient was in bed on August 28, 1911. He was awakened by a cramp in the right leg which was drawn up and flexed at knee, the thigh flexed on abdomen and he was unable to extend the lower extremity. Cramp then extended to right arm and consciousness was lost for about 15 minutes. His mother states that the left side was not affected as far as they observed.

In 1905, a short time after the first attack described above, the patient noticed a sudden jerking movement of the right arm, while employed as a telegraph operator. The arm would be thrown forward from the shoulder and he could not keep his hand on the telegraph key, and in writing, the pen would tear the paper and spoil the message.

At first the jerkings occurred only a few times daily but during the following years they became more severe and frequent and were especially so when he was excited. He had so-called "good days" and "bad days" which were directly dependent on his emotional condition. When he was able to keep quiet and had no special irritation he could go a whole day without the jerkings. Any excitement would bring on the spasms with great severity, and he found that whiskey had a quieting influence and he used this freely to keep at work on the days when they were severe. Sometimes one or two drinks would suffice for the entire day.

Physically the patient is a strong, muscular, well developed adult. Chest and abdominal viscera normal. His mental condition is very good.

There is no disturbance of the special senses nor of any of the cranial nerves. The fundus of the eye is normal and the fields normal to rough test. Pupils equal and react to light and accommodation. No disturbance of ocular movements. The facial muscles were not involved in the myoclonic jerkings during any of the examinations.

The sensory functions are normal. There is no anaesthesia, analgesia nor thermanalgesia over any part of the body. Joint sense and deep muscle pain sense normal.

The motor disturbances form the conspicuous part of the clinical picture. There is no weakness of any muscles and the range of movements is normal in all directions, and there are no contractures.

Sudden shock-like spasms occur in the muscles of the neck, shoulders, trunk, abdomen, back, thighs and arms. The spasms are bilateral, but a little more severe on the right side, and may occur involuntarily in paroxysms when patient is sitting quietly in a chair. The head is jerked backward or to one side, the shoulders and arms thrown forward, the trunk suddenly straightened or flexed, or the thighs flexed on the abdomen.

These severe involuntary paroxysmal spasms occur only on the "bad days." On the "good days" they occur only on voluntary movement, and if he is lying quietly in bed or sitting comfortably in a chair with muscles relaxed there is not the slightest spasm.

Extending the arms forward can be done slowly without bringing on a spasm, but as soon as he attempts to shake hands or to touch an object brought near the hand, the violent incoordinate jerking commences in the shoulder muscles and persists until the extremity is brought into a condition of muscular relaxation. The same condition occurs in the lower extremities. This is an emotional excitement as the mere sight of an object being brought near the hand produces the incoördinate movements independent of any voluntary effort on the part of the patient to touch it.

In testing the hand grasp an irregular rhythmical contraction and relaxation is felt in the hand and forearm and a to and fro movement from the shoulder.

Dynamometer shows right hand grasp 37 kilograms and left hand 30.

When the observer's hand is placed over the shoulder muscles during the spasms, the actual movement in the muscles is felt to be greatly in excess of that communicated to the arm. This is probably because the spasm does not involve all the synergic muscles of the shoulder but affects some in excess of the others.

At times the spasms have been so severe that patient has been confined to bed and every movement is accompanied by the violent incoördinate jerkings. When they are less severe he is able to walk in a jerky, incoördinate manner, the limbs being thrown forward or the body suddenly flexed, and on several occasions he has been injured by falling. On the "good days" he walks fairly well without assistance, but always in a cautious apprehensive manner.

In arising from a sitting position, he slides forward to the edge of the chair, places the feet widely apart and flat on the floor and then waits for a favorable intermission in the spasms to stand suddenly erect with aid of hands on thighs. Sometimes he makes several unsuccessful attempts to arise before accomplishing the act.

There is no Rombergism. When he is able to stand alone there is no increase in the swaying or muscular spasm on closing the eyes.

Slow passive movements of the extremities exert a quieting influence on the spasms. The limbs can be put into any physiological position without bringing on the spasms, and after passive exercise of this kind the incoördinate movements are less severe.

The myotatic irritability is increased and tapping a muscle brings on the spasms which involves the group of muscles and not the isolated muscle which is irritated.

All the deep reflexes are greatly exaggerated and equally so right and left. The abdominal-epigastric reflex is very active. There is no ankle clonus and no Babinski sign, and the Chaddock infra-malleolar reflex is absent.

There is no disturbance of the sphincters. The bowels are usually constipated.

Smoking or indiscretion in diet increases the muscular movements and the last epileptoid attack followed a gastro-intestinal disturbance. Alcohol has had a temporary quieting influence on the myoclonus as is usual in these cases, but he has taken none for some time.

Graduated gymnastic exercises by a competent instructor have resulted in more control over the muscular movements and patient can now perform many movements slowly without exciting the spasms.

Bromides and chloral have a temporary quieting influence but the persistent use of these or other depressants is to be avoided if possible.

The best results are to be expected from dietetic and hygienic measures and the training of the incoördinate movements by graduated and systematic gymnastic exercises.

The bibliography of Myoclonus and Myclonus-Epilepsy may be found in the following articles:

Clark, Archives of Neurology and Psychopathology, Vol. II. 1899.

Clark, Review of Neurology and Psychiatry, 1907.

Lundborg, Die Progressive Myoclonus-Epilepsie, 1903.

Hunt, J. R., Journal of Nervous and Mental Disease, 1903.

Shanahan, Ibid., 1907.

